# Towards elimination of visceral leishmaniasis in the Indian subcontinent—Translating research to practice to public health

**DOI:** 10.1371/journal.pntd.0005889

**Published:** 2017-10-12

**Authors:** Siddhivinayak Hirve, Axel Kroeger, Greg Matlashewski, Dinesh Mondal, Megha Raj Banjara, Pradeep Das, Ahmed Be-Nazir, Byron Arana, Piero Olliaro

**Affiliations:** 1 Global Influenza Programme, World Health Organization, Geneva, Switzerland; 2 Centre for Medicine and Society and Centre for Anthropology, Freiburg University, Freiburg, Germany; 3 Special Programme for Research and Training in Tropical Diseases (TDR), hosted by the World Health Organization, Geneva, Switzerland; 4 Department of Microbiology and Immunology, McGill University, Montreal, Canada; 5 Nutrition and Clinical Services division, International Center for Diarrheal Disease Research Bangladesh, Dhaka, Bangladesh; 6 Central Department of Microbiology, Tribhuvan University, Kathmandu, Nepal; 7 Rajendra Memorial Research Institute of Medical Sciences, Indian Council of Medical Research, Patna, India; 8 Department of Microbiology and Parasitology, National Institute of Preventive and Social Medicine, Dhaka, Bangladesh; 9 Cutaneous Leishmaniasis unit, Drugs for Neglected Diseases Initiative, Geneva, Switzerland; Pasteur Institute of Iran, ISLAMIC REPUBLIC OF IRAN

## Abstract

**Background:**

The decade following the Regional Strategic Framework for Visceral Leishmaniasis (VL) elimination in 2005 has shown compelling progress in the reduction of VL burden in the Indian subcontinent. The Special Programme for Research and Training in Tropical Diseases (TDR), hosted by the World Health Organization (WHO) and other stakeholders, has coordinated and financed research for the development of new innovative tools and strategies to support the regional VL elimination initiative. This paper describes the process of the TDR’s engagement and contribution to this initiative.

**Methodology/principal findings:**

Multiple databases were searched to identify 152 scientific papers and reports with WHO funding or authorship affiliation around the following 3 framework strategies: detection of new cases, morbidity reduction, and prevention of infection. TDR has played a critical role in the evaluation and subsequent use of the 39-aminoacid–recombinant kinesin antigen (rK39) rapid diagnostic test (RDT) as a confirmatory test for VL in the national program. TDR has supported the clinical research and development of miltefosine and single-dose liposomal amphotericin B as a first-line treatment against VL. TDR has engaged with in-country researchers, national programme managers, and partners to generate evidence-based interventions for early detection and treatment of VL patients. TDR evaluated the quality, community acceptance, and cost effectiveness of indoor residual spraying, insecticide-treated bed nets, insecticide-impregnated durable wall linings, insecticidal paint, and environmental management as tools for integrated vector management in reducing sandfly density.

**Conclusions/significance:**

TDR’s engagement with country policy makers, scientists, and clinicians in the development of effective diagnosis, treatment, case detection, and vector control represents an important example of TDR’s stewardship toward the elimination of VL in the Indian subcontinent.

## Author summary

Since the early days of the kala-azar elimination programme in the Indian subcontinent, TDR has engaged with national control and research institutions to conduct research aimed at informing country policy and practice to identify and treat cases and to prevent transmission of the infection. This includes the evaluation of the rK39 rapid diagnostic test for the diagnosis of VL, the clinical development and evaluation of first-line treatments (miltefosine and liposomal amphotericin B), the generation of evidence-based tools for early detection and complete clinical management of VL, the evaluation of the cost effectiveness of indoor residual spraying, insecticide-treated bed nets, insecticide-impregnated durable wall linings, and environmental vector management as tools for integrated vector management. The interaction and interdependence between implementation research, technical advice, partnership, and policy is yet another example of TDR’s stewardship contribution and empowerment toward VL elimination in the Indian subcontinent. Continuing investment in translational research from the bench to the bedside to public health, established jointly by national control programmes, academics, and TDR coordinators, is imperative to block transmission and prevent a resurgence of VL in the future.

## Introduction

About 147 million people are at risk of visceral leishmaniasis (VL), also known as kala-azar in the Southeast Asian region [1]. The largely localized geographic endemicity, anthroponotic transmission with humans as the only host reservoir, the sandfly species *Phlebotomous argentipes* as the only vector species, and the availability of effective tools for diagnosis and treatment, all supported by historical evidence for the disappearance of VL in the 1970s following insecticide spraying for malaria eradication, favour the elimination of VL as a public health problem in the Indian subcontinent [2, 3]. The World Health Organization (WHO) has identified leishmaniasis as a category I disease (emerging and uncontrolled), and the World Health Assembly (WHA) 43.18 resolution recognizes leishmaniasis as a major public health concern [4]. In 2005, the Ministers of Health of Bangladesh, India, and Nepal affirmed strong political commitment through intercountry cooperation and crossborder collaboration to eliminate VL by 2015 by reducing incidence to less than 1 per 10,000 population at the upazila, administrative block, and district levels in Bangladesh, India, and Nepal, respectively [5]. The WHA 60.13 resolution in 2007 mandates WHO to update the epidemiological evidence and take the lead in providing technical assistance in initiation, maintenance, and expansion of leishmaniasis control programmes. The VL elimination framework, further updated in 2012, identifies early diagnosis and complete case management, effective disease and vector surveillance, social mobilization and building partnerships, and clinical and operational research as 5 key strategies for achieving the elimination goal [6]. A more recent initiative led WHO to define a road map for prevention, control, elimination, and eradication of 17 neglected tropical diseases, including VL, by 2030 as a step toward achieving the Sustainable Development Goals [7]. The neglected tropical diseases road map is endorsed by donor partners and stakeholders who have pledged support to sustain national programmes, extend drugs and interventions, and monitor progress towards VL elimination by 2020 [8].

The decade following the launch of the Regional Strategic Framework for VL Elimination in 2005 has shown a substantial reduction of incident VL cases by more than 75% in the Indian subcontinent [9]. Only 16 of the 140 previously endemic upazilas in Bangladesh reported an incidence rate above the elimination target in 2013 [10], further down to 2 upazilas in 2015. An independent assessment of the national VL elimination programme indicates that all of the 12 previously endemic districts in Nepal have achieved VL elimination since 2013 and maintained the elimination status thereafter [11]. Despite considerable progress and a declining trend in the incident VL cases, 90 of the 456 endemic blocks (20%) continue to be highly endemic for VL in India [12]. On the other hand, new ecologic niches of focal indigenous transmission have emerged in hitherto nonendemic hilly areas of Nepal and Bhutan, as well as in Bangladesh and Thailand [12–15].

Since 2005, the Special Programme for Research and Training in Tropical Diseases (TDR) has coordinated and financed research for development of new innovative tools to support the VL elimination initiative in the Indian subcontinent. TDR, in conjunction with the WHO Neglected Tropical Disease group, has worked in close coordination with academia, technical and development partners, financial institutions, and the pharmaceutical industry to collaborate with regional researchers, national disease control programmes, and policy makers to identify gaps in knowledge, define research needs, and generate evidence to inform the Regional Technical Advisory Group tasked with guiding the regional and national strategy, policy, and public health practice for VL elimination in the Indian subcontinent. As countries consolidate the gains from the attack phase and transition to maintain the achievements, it is important to understand the lead coordination role of TDR and WHO to drive this elimination process.

Critical contributions by many stakeholders, including national and international actors, have supported VL elimination efforts in the Indian subcontinent—ranging from drug availability (the AmBisome donation from Gilead Sciences managed by the WHO Neglected Tropical Disease group) to support, to deployment of interventions from the Bill & Melinda Gates Foundation and the United Kingdom government, and many others. Many of these efforts were to strengthen the elimination programme and did not necessarily arise from research needs of the national programmes. This paper focuses on the knowledge generated through research, which in turn translated to practice and public health. The purpose of this paper is to describe the process of TDR’s engagement with and contribution to the VL elimination initiative in the Indian subcontinent. This paper is not intended to be a comprehensive review of VL diagnosis and treatment or an evaluation of the VL elimination programme in the Indian subcontinent. This paper brings together and critically analyses the context and the process of translating WHO TDR–supported research to effectively inform public health practice and policy as a public health model for other public health initiatives.

## Methods

We restricted our review to include published and unpublished literature (conference presentations, meeting reports) on TDR-supported research that contributed directly or indirectly towards the goal of VL elimination in the Indian subcontinent. We searched multiple databases (United States National Library of Medicine, the National Database of Indian Medical Journals) using different combinations of Medical Subject Heading (MeSH) terms, including ‘leishmaniasis, visceral’ and text words such as ‘rK39,’ ‘miltefosine,’ ‘amphotericin,’ and ‘vector control’ with and without restriction to MeSH terms ‘Bangladesh’, ‘India,’ and ‘Nepal.’ We screened each article for eligibility around 3 broad thematic areas in alignment with the Regional Strategic Framework for VL Elimination: (1) detect new cases—evaluation of diagnostic tools for case detection, strategies for early detection of new cases; (2) reduce morbidity—evaluation of drugs for VL treatment, strategies to ensure complete treatment; and (3) prevent infection and/or interrupt transmission—evaluation of vector-control strategies, role of asymptomatic infection, and post kala-azar dermal leishmaniasis (PKDL) in transmission. We included only those studies that acknowledged funding by TDR or had contributing author(s) affiliated with TDR. In addition, we searched the WHO Institutional Repository for Information Sharing (WHO IRIS) and the WHO South-East Asia Regional Office library services to identify and review policy documents, and we also searched WHA resolutions, WHO technical reports, expert consultation meeting reports, TDR annual reports, tool kits developed for VL, and Regional Technical Advisory Group meeting reports. We included expert commentaries, opinions, and reviews authored or funded by WHO TDR. In addition, we invited researchers leading WHO TDR–funded VL research in the Indian subcontinent to share preliminary findings or manuscripts in preparation for any ongoing or completed research.

## Results

The literature search yielded 104 scientific papers with acknowledged TDR funding that included 72 research studies, 18 reviews, and 14 commentaries. In addition, we retrieved 48 WHO documents, including meeting reports, technical reports, annual reports, manuals, and tool kits related to VL in the Indian subcontinent.

### Search for a field-based rapid diagnostic test for VL

The WHO established the clinical case definition for VL disease in 1996 [16]. Starting a treatment that is high on cost and toxicity on the basis of clinical suspicion alone is not justified and requires a confirmatory diagnostic test for a decision to treat [17]. However, direct demonstration of the parasite in tissue biopsies is invasive and must be done by skilled medical personnel to be done safely. Since the 1980s, TDR’s research priority has been to identify a simple, yet highly sensitive (>95%), specific (>90%), and reproducible diagnostic test that is easy to use by a front-line health worker in a field setting where the suspicion index is lower than at a referral hospital [18]. A systematic review of near-patient diagnostic tests in 1999 highlights the absence of robust standards for diagnostic trials and a need for stricter controls in procurement, introduction, and deployment of diagnostic tests in national programmes in low- and middle-income countries [19, 20].

Other methods for identifying the parasite are molecular diagnosis, which is complex and expensive [21], and the direct agglutination test (DAT), which is highly sensitive and specific but limited by its complexity and antigen variability [22–24]. Moreover, DAT production could not be sustained because of its high cost. An enzyme-linked immunosorbent assay (ELISA) based on a 39-amino-acid–repeat recombinant kinesin antigen (rK39) from *Leishmaniasis infantum* was found to be highly sensitive and specific but was not suitable for use in field conditions [25, 26]. The rK39 antigen, when introduced into an immunochromatographic strip, performs well for diagnosing active VL in field conditions [27]. TDR, in close coordination with manufacturers, evaluated several prototypes of rK39-based immunochromatographic tests (ICTs) in both the Indian subcontinent and East Africa [28–34]. The rK39-based ICTs perform consistently well with high reproducibility under field setting in the Indian subcontinent (Table 1). Based on this extensive evaluation, WHO recommended the use of rK39 ICT in the diagnosis of active VL in 2006, which was subsequently adopted by the national VL elimination programmes in Bangladesh, India, and Nepal [35]. A user guide for the rK39 rapid diagnostic test (RDT) was published by WHO in 2008 [36]. The rK39 ICT meets almost all the Affordable, Sensitive, Specific, User-friendly, Rapid and robust, Equipment-free and Deliverable (ASSURED) criteria [37]. Since then, several generic commercial versions of rK39 ICTs have emerged with anecdotal reports of the supply of counterfeit or substandard RDTs in the market. The challenge, then, was to ensure stringent external quality assurance on these rapid diagnostic kits in both the private and public sectors [38]. TDR coordinated the diagnostic performance evaluation of 5 commercially available products that were rapid (test result within 15 minutes), simple to perform with minimal equipment and training, and easy to interpret (cassette or strip format with visual readout), involving 9 testing laboratories (4 in the Indian subcontinent), which showed that all 5 commercial brands that were tested performed well in the Indian subcontinent [20, 39].

**Table 1:** TDR engagement in development research for field-based RDT for detection of visceral leishmaniasis in the Indian subcontinent.

Author Year Country Reference	Year/Extent of TDR Engagement	Study Design Subjects Sample Size	Results	Conclusion
Chappuis 2003 Nepal [30]	1999–2000 Funding Authorship	Diagnostic evaluation (rK39 ICT, DAT) study 184 VL patients	rK39 ICT sens– 97%, spec– 71%; DAT sens– 99%, spec– 82%;	rK39 ICT compares well with DAT; easy to use in field setting; rK39 ICT can be used for screening test for VL and as a confirmatory test for VL only in high prevalence VL areas due to its high PPV
Boelaert 2004 Nepal [28]	2000–2002 Authorship	Diagnostic (rK39 ICT, FGT, IFAT, DAT) evaluation study 310 VL patients	rK39 ICT sens– 87.4%, spec– 93.1%; FGT sens– 39.9%; IFAT sens– 28.4%; DAT sens– 95.1%	DAT, rK39 ICT can replace parasite diagnosis by bone marrow or splenic aspirate as basis for decision to treat VL in national VL elimination programme
Chappuis 2006 Nepal [31]	2001–2002 Funding Authorship	Diagnostic (rK39 ICT, FGT, KAtex) evaluation study 85 VL patients	rK39 ICT sens– 89%, spec– 90%; FGT sens– 52%; KAtex sens– 57%; Reproducibility higher for rK39 ICT (κ = 0.87) compared to FGT and KAtex;	rK39 ICT meets most criteria of ASSURED [37]
Sundar 2007 India [34]	2005 Funding	Diagnostic (rK39 ICT, rK26 ICT, DAT-FD, KAtex) evaluation study 282 VL patients	rK39 ICT sens– 98.9%, spec– 97%; DAT-FD sens– 98.9%, spec– 94%; KAtex sens– 67%;, spec– 99%; rk26 ICT sens– 21.3%, spec– 100%; Reproducibility high (κ>0.94) for all tests; High agreement between rK39 ICT and DAT-FD (κ = 0.986);	rK39 ICT easy to use in field and preferred RDT for VL elimination programme
Boelaert 2008 India, Nepal, East Africa [29]	2003–2006 Funding Authorship	Diagnostic (rK39 ICT, DAT-FD, KAtex) evaluation study 1,150 VL patients	rK39 ICT, DAT-FD sens > 96%, spec– 90%; DAT-FD sens– 98%, spec– 91%; KAtex sens– 35–66%;, spec– 87–97%; Reproducibility high (κ > 0.94) for DAT-FD, rK39 ICT	DAT-FD, rK39 ICT performance variable and lower in East Africa; DAT-FD, rK39 ICT recommended for clinical practice in Indian subcontinent
Mohapatra 2010 India [26]	Funding	Diagnostic (rK9, rK26, rK39, CSA, ELISA) evaluation study 55 VL patients	rK39 sens– 100%, spec– 96% rK9 sens– 78%, spec– 84% rK26 sens– 38%, spec– 80% CSA sens– 80%, spec– 72%	rK39 most suitable antigen compared to rk9, rk26, CSA; rK9 antigen may be used as adjunct to rK39 for accurate diagnosis of VL or if rK39 antigen not available
WHO 2011 ISC, East Africa, South America [20]	2009 Funding Authorship	Diagnostic (5 commercial RDTs—rK39 ICT, rkE16 ICT) evaluation study 250 VL patients 9 testing laboratories (4 in Indian subcontinent)	Accuracy of RDTs between centres comparable but significantly different between regions; sens, spec, reproducibility (operator to operator, run to run), heat stability high for all RDTs in Indian subcontinent, variable in East Africa, South America	In Indian subcontinent, all brands of RDTs performed well; Need to establish minimal performance limits; Results can be used to guide procurement.
Cunningham 2012 ISC, East Africa, South America[39]	2009 Funding Authorship	Diagnostic (five commercial rK39 ICT) evaluation study 550 VL patients	All rK39 ICTs good sens (92.8–100%) and spec (96–100%) in Indian subcontinent; Lower and variable sens in East Africa and South America; Reproducibility (operator to operator, run to run) high (κ = 0.73–0.99)	Commercial rK39 ICT kits performed well in Indian subcontinent; Need to assess performance in HIV-compromised VL patients
***Reviews***				
Sundar 2002 ISC [24]	Funding	Review	Parasite diagnosis by splenic or marrow or skin lesion remains gold standard but with limitations; DAT limited by cost, multiple steps, incubation, and antigenic variation;	rK39 ICT good sens and spec, rapid results, and can be used in field setting; Need R&D for urine-based KAtex and field-adaptable version of PCR.
Boelaert 2007 [18]	Authorship	Review of considerations for evaluation of diagnostic tests (test for case detection, cure, relapse, surveillance, drug resistance, certification of elimination)	High performance of rK39 ICT (InBios) in India [32]; lower spec (71%) in Nepal in early prototype; higher spec [30] in later generation of InBios ICT [28] and with DiaMed ICT [40];	Need to standardize methodology for evaluation of RDTs to prevent substandard or counterfeit products being used in endemic areas.

Abbreviations: CSA, crude soluble antigen; DAT-FD, direct agglutination test, freeze-dried antigen; FGT, formol gel test; ICT, immunochromatographic card test; IFAT, immunofluorescent antibody test; ISC, Indian subcontinent; KAtex, latex agglutination test for leishmania antigen; R&D, research and development; RDT, rapid diagnostic test; rK9, recombinant kinesin 9; rK26, recombinant kinesin antigen 26; rK39, recombinant kinesin antigen 39; sens, sensitivity; spec, specificity; VL, visceral leishmaniasis.

Undoubtedly, the rK39 ICT has been an essential tool in the elimination programme. To identify active VL, it is used as one element of the diagnostic algorithm whereby it is applied to subjects with persistent fever and palpable spleen. By itself, it cannot differentiate active from past infection [41], and it has limited value as a marker for disease progression, cure, or relapse [18]. The search continues for a new diagnostic marker that can be used at the population level—a marker for asymptomatic infection, for progression to PKDL, that performs well even in VL–HIV-coinfected individuals [42]. Notwithstanding these shortcomings, the rK39 ICT story is a fine instance of how a public–private partnership between TDR, country-based researchers, national programme managers, and industry can move a product intended for a neglected disease affecting the poorest of poor from the bench to the bedside to public health practice and ensure sustained availability in a short time with focused funding.

### Quest for a safe effective and affordable alternative for VL treatment

Pentavalent antimony has been the mainstay for VL treatment for more than 6 decades, despite its toxicity, need for parenteral administration in a healthcare setting, and a long course of therapy. Reports of increasing treatment failure rates of up to 65% in Bihar, India in the 1980s and 1990s [43, 44] spurred the search for an alternative treatment. TDR was involved in the development and evaluation of various treatments for VL, and pioneered new solutions. TDR was ahead of its time in supporting the development and registration of miltefosine through an early form of public–private partnership, supported the extension of indication of liposomal amphotericin B for VL, initiated the development of Paromomycin for VL, and pioneered combination regimens for VL.

The serendipitous laboratory discovery in the mid-1980s of miltefosine, an anticancer drug, against the *Leishmania* parasite in vitro and after oral use in animals [45] focused interest on the potential of miltefosine to replace pentavalent antimonial as a first-line drug against VL. Clinical trials were supported jointly by TDR, and the then-manufacturer Asta-Medica/Zentaris showed that miltefosine was safe and efficacious (more than 90% cure rate) in adults and children (Table 2, S1 Appendix) [46–49]. Miltefosine was then registered as the first oral treatment for VL in India in 2002 and subsequently introduced into the national VL elimination programme in 2006 following a TDR-supported phase IV trial that tested the feasibility of miltefosine use in an outpatient setting [50]. After a phase of deployment during which miltefosine contributed successfully to improving VL case management in the context of the VL elimination programme, miltefosine is no longer widely used. This is largely because the preferred first-line treatment has become single-dose liposomal amphotericin B, which overcomes some of the challenges associated with the use of miltefosine—its teratogenic potential that requires women of reproductive age to take contraceptives and causes issues with compliance as a result of the month-long therapy, which could potentially result in drug resistance. Moreover, despite binding agreements signed between WHO and the manufacturer to secure its affordability [51], miltefosine is not widely available as a consequence of low production and high prices.

**Table 2:** TDR-funded and/or TDR-supported drug development research towards elimination of visceral leishmaniasis in the Indian subcontinent.

	Miltefosine	Liposomal Amphotericin B	Paromomycin	Combination Therapy
1995–1999	India (1999): Phase II trial (100 mg/d x 28 d)–Cure rate (97% at 6 mo) [48]	India (1996): Phase II trial–Cure rate (100% at 12 mo); High efficacy, safe [52, 53]	India (1998): Phase II trial–Cure rate (16 mg/kg x 21 d– 93%, 20 mg/kg x 21 d– 97% at 6 mo); preferred first-line treatment in areas of SSG resistance [54]	India: Phase II trial (PM 12 mg/kg + SSG 20 mg/kg) x 21 d more effective (cure rate 88%), safer than SSG (20 mg/kg x 40 d) in areas of SSG resistance [55]
2000			India: Phase II trial–Cure rate (12 mg/kg x 21 d– 90%, 16 mg/kg x 21 d– 89%, 20 mg/kg x 21 d– 86% at 6 mo); preferred first-line treatment in areas of SSG resistance [56]	India: Phase II trial (PM 12 mg/kg + SSG 20 mg/kg) x 21 d more effective (cure rate 92%), safer than SSG (20 mg/kg x 28 d, cure rate 53%) in areas of SSG resistance [57]
2002	India: Phase III trial (100 mg/d x 28 d)–Cure rate (94% at 6 mo) similar to AmphB [46]			
2003	India: Phase I/II trial (2.5 mg/kg x 28 d)–Safe, cure rate (90% at 6 mo) in children [47]			
2004	India: Phase I/II trial (2.5 mg/kg x 28 d)–Safe, cure rate (94% at 6 mo) in children [49]	India: Phase III trial–Cure rate (L-AmB 96%, Abelcet 92% at 6 mo) similar to AmphB, better tolerated, shorter therapy (5 d), less hospitalization cost [58]		
2006		WHO guideline for L-AmB as first-line treatment in areas of drug resistance and VL coinfection with HIV [59]		
2007	India: Phase IV trial–Cure rate (82% at 6 mo) in outpatient setting [50]			
2008				India: Phase II trial–L-AmB at single reduced dose (3.75 mg/kg) + miltefosine short duration (7 d) is highly efficacious (cure rate at 6 mo– 98%) [60]
2011				India: Phase II trial combination therapy (L-AmB single dose + miltefosine–cure rate at 6 mo 98%; or with PM–cure rate at 6 mo 99%) more effective than monotherapy with AmphB (cure rate at 6mo– 93%) [61] India: Phase III trial–L-AmB single dose + miltefosine x 14 d cure rate– 92% [62]
2014		Bangladesh: Phase III trial (10 mg/kg x 1 d)–Safe, cure rate (97% at 6 mo) in PHC setting [63, 64]		

Abbreviations: AmphB, amphotericin B; L-AmB, liposomal amphotericin B (AmBisome); PHC, Primary Health Care; PM, Paromomycin; SSG, sodium stibogluconate.

Amphotericin B dexoycholate, a systemic antifungal, despite being toxic and requiring slow intravenous infusion, has been in use for more than 2 decades as a second-line drug for VL. The liposomal formulation of amphotericin B (L-AmB), however, could be delivered as a short-course treatment and was much less toxic than other therapies. Consequently, dose-finding studies for L-AmB funded by TDR [52, 53, 58] supported the extension of registration of AmBisome and the WHO recommendation of L-AmB as the first-line treatment for VL in the Indian subcontinent [59]. Subsequent studies of L-AmB were undertaken with the support of the Drugs for Neglected Diseases initiative (DNDi) and Médecins sans Frontières (MSF). The high cost of AmBisome, along with the need for 3–5 intravenous infusions, however, initially restricted the rollout of L-Amb to referral facilities [65]. In 2007, WHO secured a preferential price with the manufacturer Gilead Sciences for AmBisome to be available for the Indian subcontinent at 10% of the original retail price for low- and middle-income countries. This prompted a landmark study by Sundar et al. that showed a high efficacy of more than 95%, even with a single dose of 10 mg/kg L-Amb [66]. A phase III trial in Bangladesh supported by the WHO Neglected Tropical Disease group further established the safety and effectiveness of treating VL with a single-dose L-AmB in a secondary healthcare facility (Upazila Health Complex) [63], and a TDR-supported study demonstrated its feasibility and acceptance [64]. In 2010, WHO negotiated a donation of up to 445,000 vials of AmBisome at the preferential price for the Indian subcontinent to cover the predicted case load to 2016 and as required to 2021 [7]. Consequently, single-dose AmBisome replaced miltefosine as the first line of treatment in the national VL elimination programme in the Indian subcontinent [67].

The clinical development of Paromomycin, an aminoglycoside antibiotic with anti-*Leishmania* properties, has been slow. TDR initiated the phase II trials of Paromomycin in the early- and mid-1990s [54–57, 68]. Further development of Paromomycin was halted as attention focused on miltefosine. The interest in Paromomycin picked up again after the institute of OneWorld Health (iOWH) conducted the pivotal phase III trial, which supported registration [69]. The parenteral formulation of Paromomycin used in the clinical trials was no longer available, and the registration of the new formulation was delayed until 2006.

As is the case for other infectious organisms, single-agent treatments can select for resistant *Leishmania* parasites. Learning from other diseases, TDR pioneered the study of combination regimens. In the mid-1990s, TDR-supported trials demonstrated that the loss in efficacy of sodium stibogluconate (SSG) in SSG-resistant areas was overcome by combining it with Paromomycin [55, 57]. Later, this combination was further studied by the DNDi in East Africa. More than a decade later, following reports of miltefosine treatment failures [49, 70, 71], TDR collaborated with Indian researchers to conduct the first dose-finding trial of single-dose L-Amb plus miltefosine combination [60]. This study informed a subsequent larger trial of different combinations of L-Amb, miltefosine, and Paromomycin supported by the DNDi [61, 62]. Coadministering drugs has the advantage of reducing dosage and toxicity, shortening treatment duration, improving compliance, and reducing the chance of resistance to individual drugs, thus potentially prolonging a drug’s lifespan of effective use [72]. TDR contributed to various studies supporting the use of combination therapies and providing evidence that they are more cost effective and avert more deaths and years of life lost than monotherapies [65, 72–75]. Combination therapy, however, requires strict supervised deployment to avoid the erosion of efficacy due to subtherapeutic dosing practices [76], and there is a need to monitor prescribing practices, the knowledge of health care providers, drug availability, quality, and safety through pharmacovigilance [77].

### Understanding VL as a public health concern in the Indian subcontinent

The WHO has periodically reviewed the global burden of leishmaniasis since the early 1990s and estimates that more than 90% of the global burden of VL was in 6 countries (India, Bangladesh, Sudan, South Sudan, Ethiopia, and Brazil) [78–81]. A new country leishmaniasis profile created in 2010 in the WHO Global Health Observatory Data Repository monitors the endemic status of countries and trends in the number of reported VL cases since 2005 [82]. TDR-supported research showed a more than 8-fold underestimation of disease burden [83, 84], an annual VL incidence that was up to 22 times higher than the elimination target [85], a 6% case fatality rate (Table 3) [86], and established a baseline for the attack phase of the VL elimination programme. Furthermore, TDR-supported epidemiological research defines and corroborates the role of poverty, caste, literacy, housing condition, proximity to vegetation, water bodies, livestock, and sleeping habits in influencing exposure to the risk of VL, which helped understanding of human–vector transmission and to inform vector-control strategies for VL elimination [87–92].

**Table 3:** Understand the epidemiology: TDR-supported and/or TDR-authored research for elimination of VL in the Indian subcontinent.

Early Detection	Complete Treatment	Vector Control
**Phase 1: Understand the epidemiology** [14, 78, 79, 83, 85–91, 93–96]		
- What is the VL burden? - How much does a passive reporting underestimate the VL burden? - What are the risk factors? - Are there delays in diagnosis of VL?	- What is the community’s KAP about VL? - Are there delays in seeking treatment for VL?	- What vector-control measures are in use? - What is the community awareness on vector control for VL? - How is the quality of IRS in India, Nepal?
- Disease burden estimates based on passive surveillance; mortality data sparse based on hospital deaths - VL case fatality rate (6.12%) 17 times higher in tribal population in Bangladesh - Annual incidence up to 22 times higher than elimination target in Indian subcontinent - More than 8-fold underreporting - Poverty impedes early diagnosis and treatment, increases risk to VL; VL in turn reinforces poverty - Low literacy, low caste, large families, poor housing, proximity to water, vegetation, livestock, and sleeping habits increases risk of VL - Delay in seeking care 3.75 times more in Nepal (30 days) than in India - Delay in diagnosis after seeking care 3.6 times more in India (90 days) than in Nepal - Delay in reporting to health system more in Nepal (76 days) than in India (28 days)	- High awareness of VL except in Bangladesh - Provider choice: formal and informal private medical practitioners (India); chemist shops and health centres (Nepal); health centres (Bangladesh) - Long delays in diagnosis and start of treatment; provider shopping by patient before availing treatment in public sector (India) - No delays from diagnosis to start of treatment in India, Nepal	- Low community awareness on VL prevention through vector control - Very limited IRS but high community use of bed nets in Bangladesh - IRS spraying substandard, suboptimal insecticide bioavailability on sprayed surfaces, SF resistance to DDT widespread (India), SF susceptible to pyrethroids (Nepal)
**Phase 2: Validate the elimination strategy** [85, 97–105]		
- Does ACD increase yield of new VL cases? - Does ACD reduce delays in diagnosis and treatment of VL? - How much effort and cost to find an undetected case through ACD? - Is it cost effective to combine ACD for VL, PKDL with vector control? - Can community participation strategy enhance detection of PKDL cases?	- Can improved drug management at health centre improve patient satisfaction, reduce treatment delay, and strengthen compliance?	- What is the efficacy of different vector-control tools? - Is ITN efficacious and acceptable in Bangladesh? - Is DWL vector-control method safe, efficacious in Bangladesh?
- Active house-to-house screening identifies 20% to 100% more VL cases depending on the endemicity levels among districts - ACD results in patients spending less for diagnosis and correct treatment - ACD (house screening) is cost effective in districts with poor surveillance systems - Effort and cost to detect new VL case through ACD increases as VL incidence decreases - Combining camp (fever, skin lesions) with ITN strategy is cost effective in detecting new cases of VL, PKDL, tuberculosis, leprosy, and malaria and reducing SF density by 86% (India), 32% (Nepal) at 4 weeks - Focal search around 32 VL patients detected 19 new VL patients - ACD of PKDL by trained community health volunteers trained in screening individuals with skin lesions suspected 52 cases, of which 9 were confirmed as PKDL on PCR	- Treatment of patients hampered by shortage of first-line drugs in India and Nepal; delay in procurement of miltefosine in Bangladesh - Positive experience with drug management at PHC level and patient satisfaction	- IRS significantly reduced SF density in research setting, LLIN and EVM less and variably effective - IRS (DDT in India, alpha cypermethrin in Nepal) effectiveness is low when implemented by the national program - ITN is highly efficacious even at 6 months; highly acceptable and feasible, less dependent on skilled staff, strong on community involvement - DWL most effective, durable, acceptable but more costly vector-control method, followed by ITN and EVM
**Phase 3: Compare approaches** [73, 74, 105–112]		
- Which diagnostic strategy is most cost effective for VL treatment? - Which is the most cost-effective ACD approach?	- What are the constraints and benefits of delivering home-based treatment with oral miltefosine? - Does home-based treatment with oral miltefosine improve patient management, compliance, and satisfaction? - How does the cost effectiveness of combination therapy compare with mono therapy?	- What is the most effective vector-control strategy? - What is the comparative cost of intervention? - How do LLIN with different insecticides compare for efficacy in Nepal? - Is DWL cost-effective method for vector control?
- Clinical criteria combined with serology most cost-effective diagnostic strategy to treat VL - Blanket search: high yield but requires high effort, expensive and difficult to sustain - Camp search: optimal for high endemicity districts - Focal search: optimal for low to moderate endemicity areas - Incentive-based approach: high yield but may not be acceptable to national health system	- Performance of primary HCP in patient management is still hampered - Patient satisfaction with VL treatment in public sector is reasonable - PM least expensive treatment option, cost per YLL or death averted least for PM (US$2–US$53) and highest for L-AmB (US$22–US$527)	- IRS most effective strategy, LLIN promising alternative in Nepal, Bangladesh - LLIN significantly efficacious even after 18 months of use - IRS (India), ITN less expensive than EVM, delivery costs low, costs sensitive to cost of material (bed net, insecticide) - DWL (reduced surface area) safe, efficacious, cost-saving option for vector control compared to DWL (full surface area) - IRS combined with ITN more effective than IRS or ITN alone; acceptance higher
**Phase 4: Translate research to practice and public health** [11, 106, 111, 113–115]		
- Is it feasible, acceptable, and cost effective for national VLEP to scale up ACD appropriate to the endemicity level of VL? - What is the additional cost and human resource requirement for ACD to be scaled up by national VLEP? - What aspects of the VLEP need to be strengthened?	- What are constraints of patient management at PHC and at home for improved health services performance?	- What are the performance indicators to assess IRS? - How can quality of IRS in national programme be improved? - Is community-based intervention with ITN effective in reducing VL in Bangladesh? - Is IRS effective in India and Nepal when delivered by national programmes?
- National programme can adapt camp, focal search ACD strategies but require adequate time and resources for planning, training, and strengthening referral - ACD strategies can be scaled up by national programme with current staff with training; scale up easier if all staff positions filled - Need to strengthen disease and vector surveillance, ACD strategies, ITN, IRS, supply of drugs and RDTs, develop innovative BCC activities, resources for vector control (Nepal)		- Monitoring and evaluation tool kit for IRS developed and validated to detect constraints in IRS operations and trigger timely response - Hand compression pump easier to use, lower weight, lower operation cost, safer, higher spray coverage area, more efficient than stirrup pump - Community intervention with ITN reduced VL incidence by 66.5%

Abbreviations: ACD, active case detection; BCC, behavioral change communication; DDT, dichloro-diphenyl-trichloroethane; DWL, durable wall lining; EVM, environment vector management; HCP, health care provider; IRS, indoor residual spraying; ITN, insecticide treated nets; KAP, knowledge attitude practice; L-AmB, liposomal amphotericin B (AmBisome); LLIN, long lasting insecticide nets; PCR, polymerase chain reaction; PHC, primary health center; PM, Paromomycin; RDT, rapid diagnostic test; SF, sand fly; VLEP, Visceral Leishmaniasis Elimination Programme; YLL, years life lost.

### Early case detection and complete clinical management

A major multicentre research project was initiated by TDR in the Indian subcontinent to inform the early case detection and complete case management strategy of the regional framework for VL elimination [5]. A situational analysis indicated that community and healthcare provider awareness of VL was high except in Bangladesh [93]. There were significant delays in seeking care (30 days in Nepal, 8 days in India), long periods between seeking care and diagnosis (up to 90 days in India), and delays in reporting to the health system (up to 76 days in Nepal, 28 days in India) [96]. Health-seeking behavior patterns differed—patients in India typically sought care early on in their illness, sought care from multiple providers in both the informal and formal sectors, and remained undiagnosed and inappropriately treated for long periods. In Nepal, when patients first sought care at the health centre, they presented with the clinical signs and symptoms of VL and had a shorter duration between seeking care and diagnosis and the start of treatment.

Twenty to 100% of new, hitherto undiagnosed VL cases were detected in a house-to-house search depending on the endemic level in the district (Table 3) [85]. The detection of new cases is higher for highly endemic districts that had weak surveillance systems based on passive reporting. The effort and cost of active case detection increases in areas with a lower burden of VL. Active case detection results in patients spending less for diagnosis and treatment overall. Four different approaches for active case detection—camp, index case (focal search), incentive based, and blanket search—were compared for the yield of new cases, cost, and feasibility (Table 3) [97]. The blanket approach (screen for VL, all houses in a community) yields the most cases but is expensive, requires high effort, and is difficult to sustain but useful in VL outbreaks to search for secondary cases. The camp approach (screen for VL, all patients with fever attending a camp) is cost effective (in terms of being affordable for the control programme and being effective in identifying new cases early before they spread the parasites within the community and beyond) and suitable for highly endemic areas. The index case-based or focal search (screen for VL, all houses within a 50- to 100-m radius of a known VL case) is cost effective in low endemicity areas [104]. The incentive-based approach (healthcare providers are provided a monetary incentive for detecting a new VL case) is cost effective but may not be accepted by some health systems [105]. In assessing these approaches, it was clear that there is no single universally applicable solution, and countries selected the active case detection approaches that were suited to their endemicity level and healthcare resources and capacity [111]. In order to facilitate the implementation of these strategies, TDR developed standard operating procedures for the different case detection approaches. Based on these studies, in 2010, the Regional Technical Advisory Group recommended the use of camp and index case-based search approach by national VL elimination programmes in high- and low-endemicity areas [67]. A human resource assessment study indicated that active case detection strategies can be scaled up by national programmes in Bangladesh, India, and Nepal with current staffing levels, albeit with some training, though scale-up would be easier if all staff positions were filled [114]. A recent exploratory study showed that the combined screening of patients with fever or skin lesions for VL, malaria, tuberculosis, PKDL, and leprosy, followed by insecticide treatment of bed nets in the community, is a promising, cost-effective approach in the maintenance phase of the VL elimination programme [101].

### Interruption of human–vector transmission

A second multicentre research project supported by TDR was conducted to inform the integrated vector-management strategy of the Regional Strategic Framework for VL Elimination. A situation analysis in Bangladesh indicated that there was low community awareness that VL was transmitted through the bite of sandflies and could be prevented by vector control [94]. Indoor residual spraying (IRS) using DDT in India was substandard, and the insecticide bioavailability on sprayed surfaces was suboptimal. The sandfly was widely resistant to DDT used in India but susceptible to pyrethroids used in Nepal and Bangladesh [95]. There were no vector-control activities in Bangladesh, but community use of bed nets was high [94]. A TDR-supported study demonstrated that the hand compression pump was more user friendly, weighed less, was easier to operate, had a lower operation cost, and was more efficient with a higher discharge rate and coverage of surface area than the stirrup pump used in the Indian control programme [113].

The WHA 50.13 resolution and the Stockholm Convention calls for a reduced reliance on chemical pesticides, specifically DDT for vector control. Viable alternate strategies are needed for controlling vector-born diseases. As part of the integrated vector management, TDR research tested 3 interventions—IRS, insecticide-treated bed nets, and environment management (EVM). IRS and, to a lesser and more variable extent, EVM (lime–mud plastering of walls), and long-lasting insecticide nets (LLINs) significantly reduced sandfly density [99, 116]. Cost studies showed that IRS (in India) and LLIN are cheaper options for vector control, whereas EVM should be a voluntary and complementary option [107]. Community involvement in the dipping of bed nets in slow-release insecticide K-O Tab 1-2-3 was feasible, acceptable, and more cost effective than LLIN in reducing sandfly density and VL disease burden in Bangladesh [98, 117]. It has an operational advantage over IRS because it is less dependent on skilled personnel, climate conditions, and political commitment. The insecticide residue and bioefficacy of LLINs was shown to be high at 18 months even after 2 washes [108]. Overall, IRS is the most effective option if applied properly and needs to be adapted to seasonal variations in sand fly density. LLIN is complementary and the most effective alternative to IRS when the transmission intensity is low. The strategy to achieve a rapid and sustained reduction in sandfly density by IRS, followed by widespread distribution and use of LLIN to prevent transmission when the sand fly density rebounds, needs to be tested [109]. A recent study showed that IRS combined with ITN was more effective in reducing sandfly density, had better bioavailability over a 12-month period, and was better accepted by the community [112].

**Fig 1:**
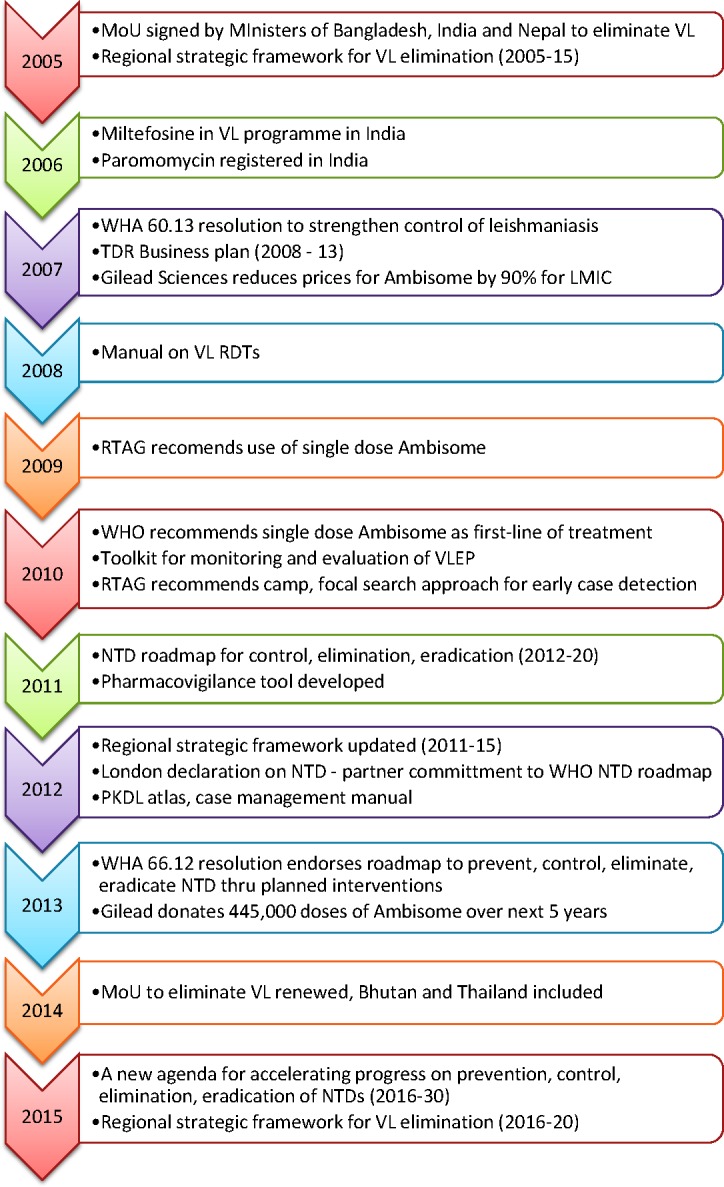
Strategic milestones achieved after adoption of the Regional Strategic Framework for Visceral Leishmaniasis Elimination in the Indian subcontinent.

Based on its ongoing research, TDR developed and field tested a monitoring and evaluation tool kit for IRS with indicators to monitor inputs (planning, training, availability of equipment, insecticide), process (spraying performance, insecticide used), output (coverage, bioavailability), outcome (sandfly density), and impact (VL disease burden) (Table 3) [118]. The tool kit was useful for detecting operational constraints in IRS, such as inadequate training of spraying squads, supervisors, deficient equipment, poor spray performance, limited surface coverage in households, etc., and triggering a timely response [106]. The Regional Technical Advisory Group recommended the adoption of the TDR monitoring and evaluation tool kit for IRS by the national VL elimination programmes in 2013 [119]. More recently, the TDR supported a multicountry study to evaluate durable wall lining (DWL) (ZeroVector; Vestergaard Frandsen, Lausanne, Switzerland)—a thin, polythene material impregnated with deltamethrin for lining the walls. Compared to ITN (bed nets impregnated with K-O Tab 1-2-3) and EVM (household walls washed with lime, sandfly breeding places treated with bleach), sandfly mortality and reduction in sandfly density was highest with DWL. DWL was most effective, durable, acceptable, and long-lasting, though more expensive, than any of the other interventions [100]. A follow-up study showed that DWL applied to a reduced surface area of the walls (1.5 m instead of 1.8 m height from the floor), had similar high efficacy, and was a cost-saving intervention [120]. The key milestones for the regional VL elimination initiative are summarized in Fig 1. The critical contributions by TDR to the VL elimination initiative are summarized in Table 4.

**Table 4:** TDR contributions to VL elimination in the Indian subcontinent.

TDR Research	Main Findings	Policy Change / Implication
Evaluation of rK39 ICT as confirmatory test for VL	Sensitivity >95% Specificity >90% Reproducibility high in field setting	rK39 ICT replaces splenic aspiration as confirmatory test for VL diagnosis and incorporated by national VL elimination programme
Miltefosine trials	Highly effective, well tolerated; feasible to administer at home under supervision of health worker	Miltefosine registered for VL; introduced as first-line treatment for VL in national program
Single-dose liposomal amphotericin-B trial	Highly acceptable and feasible when introduced at the primary health centre level	Introduced as first-line treatment for VL in national programme
Combination therapy trials	Single-dose liposomal amphotericin-B combined with miltefosine highly effective and well tolerated	Treatment policy implication during maintenance phase of VL elimination
Intervention trials to compare different strategies for early detection of VL and PKDL	Camp approach cost effective in high endemic areas; index case search approach cost effective in low endemic areas	Camp and index case search approach adapted by national VL elimination programme
Evaluation of vector-control strategies (IRS, insecticide treated bed nets, durable wall lining with insecticide) for VL elimination	IRS effective in high transmission areas; LLIN complements IRS in low-transmission areas	Integrated vector management considered as strategy for VL elimination
Development of M&E tool kits for indoor residual spraying and the VL elimination programme Research capacity building in countries affected by VL	Highlighted challenges in implementation and identified areas for improvement	M&E tool kit adapted by national VL elimination programme

Abbreviations: ICT, immunochromatographic card test; IRS, indoor residual spraying; LLIN, long lasting insecticide nets; M&E, monitoring and evaluation.

## Discussion

The VL regional elimination initiative has been a daring, cooperative endeavour that has been possible because key tools and interventions were available and could be implemented. The elimination target is now either reached or within reach thanks to these instruments and the contributions of many actors at the national and international level. TDR-supported research has played a critical role in contributing to the development and selection of the essential diagnostics and treatments (rK39, miltefosine, L-amB, Paromomycin, combination treatments), to the development of strategies and approaches to identify cases and prevent transmission in different epidemiological settings (fever camp, index case search, innovative vector-control strategies), which have been adopted and rolled out by national programs, and to the development of tools to monitor the quality and impact of the VL elimination program. As countries rapidly progress toward VL elimination, TDR now focuses on transmission dynamics and integrated approaches that are feasible and sustainable in the long term to prevent the resurgence of VL.

### The TDR model

The overall scope of TDR is to support research to develop and validate cost-effective interventions and strategies for VL elimination in the Indian subcontinent while promoting country empowerment and research capacity through the training of dozens of in-country researchers and through learning by doing [121]. TDR’s unique strategic approach to reduce the burden of illness among poor people in low- and middle-income countries is through building local, regional, and global partnerships, long-term commitment to mentoring and strengthening in-country research institutes and networks, and a downstream emphasis on intervention research to inform policy and programme implementation.

**Fig 2:**
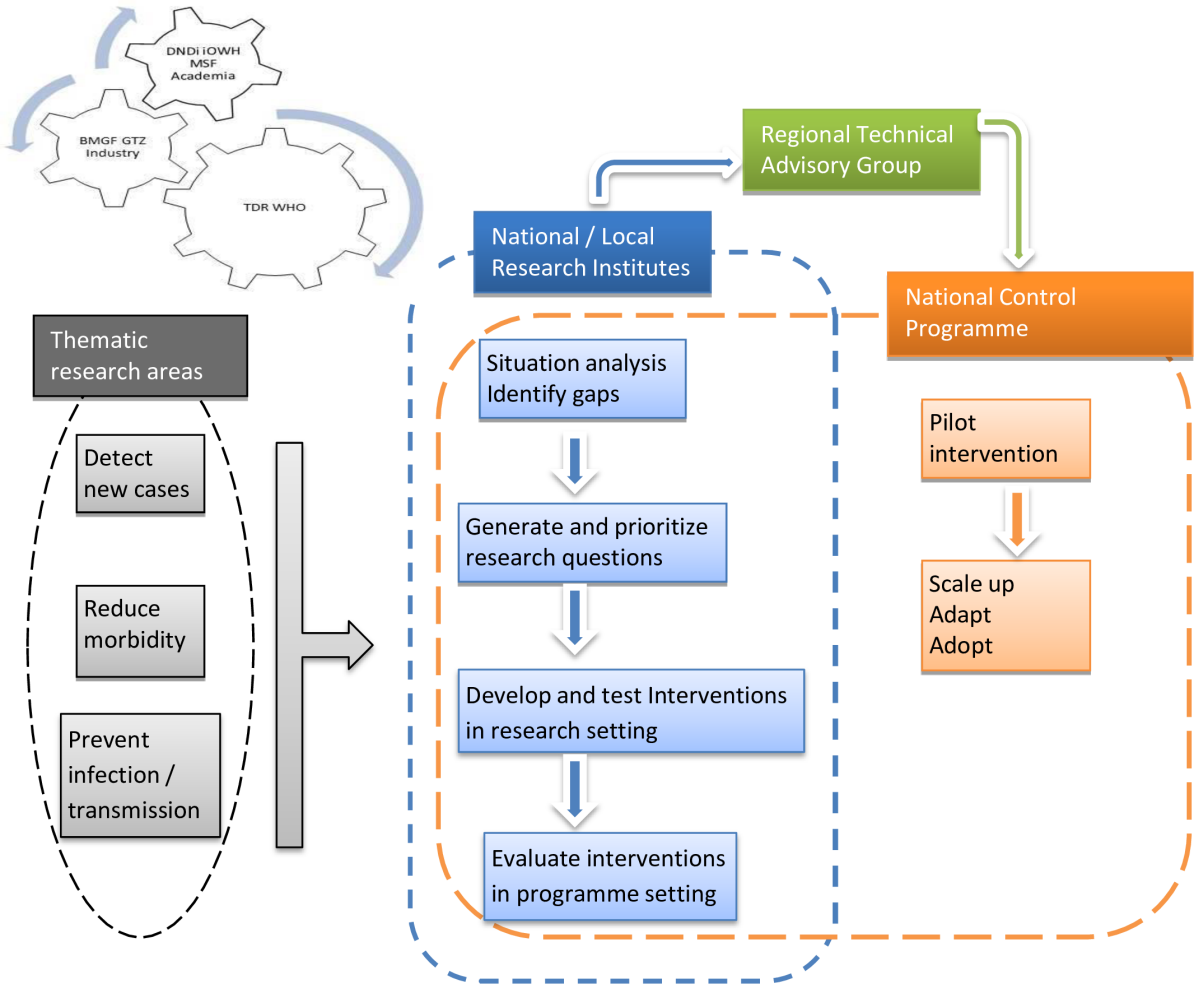
Research and development model adopted by TDR for the elimination of visceral leishmaniasis in the Indian subcontinent.

WHO created a regional policy environment and political commitment conducive for the elimination of VL from the Indian subcontinent. TDR’s approach has been to create a partnership with research institutes (the International Center for Diarrheal Diseases Research, Bangladesh [icddr,b] in Bangladesh, Rajendra Memorial Research Institute of Medical Sciences [RMRI] in India, Kala-Azar Medical Research Center [KAMRC] in India, Institute of Medicine [IOM] in Nepal, and B.P.Koirala Institute of Health Sciences [BPKIHS] in Nepal) and the control programmes of the 3 countries of the Indian subcontinent. Established in 2005, this collaboration with country-based researchers, national control programme managers, and other partners identified 3 broad thematic areas—detect new cases at an early stage, reduce morbidity, and prevent infection—for targeted intervention research aligned with the Regional Strategic Framework (Fig 2). A situational analysis defined the status of control activities and identified gaps that helped to develop and prioritize research questions. Intervention research was designed jointly in workshops with researchers and programme managers from all 3 countries. The intervention and data quality was monitored by an external monitor through site visits. Data were analysed jointly by the researchers and programme managers, and salient findings were presented to policy makers specially invited on the last day of the workshop. Tools were developed and validated to monitor and evaluate the scale up, adaption, and adoption of the interventions from a research setting into the real-life national programme setting. Linkage with the national programmes from the conception stage itself, to identify and prioritize research needs, facilitated the uptake of evidence-based interventions into national programmes and policy. The interaction and interdependence between intervention research (TDR), technical advice (Regional Technical Advisory Group), and policy (VL Elimination Programme) is yet another example of TDR’s stewardship contribution towards VL elimination in the Indian subcontinent. TDR has worked alongside other partners (DNDi, iOWH, MSF, academia, and industry) with support from the Japanese International Cooperation Agency (JICA), World Bank, Grand Challenges Canada, and Gesellschaft für Technische Zusammenarbeit (GTZ) to evaluate new diagnostics and drug treatments for VL. TDR-supported research complemented the research and development of VL drug and vector control by other partners. Moreover, WHO negotiated with industry for preferential pricing of miltefosine and AmBisome to facilitate uptake by the national programmes.

### The road ahead—Maintenance phase

As countries in the Indian subcontinent progress towards the elimination goal in the affected regions, the concern is that elimination may be mistaken for eradication, and both donor fatigue and programme complacency may drift attention to the next unfinished agenda [122]. Limitations of the current programme and the need to maintain and consolidate gains has already been highlighted elsewhere by us, and TDR remains committed to supporting operational and implementation research to achieve the elimination goal [122, 123]. The challenge now is to ensure that the disease does not reemerge or is not reintroduced and that disease and vector surveillance are reinforced during the postelimination phase [124]. The strategy needs a paradigm shift from preventing disease to preventing infection and interrupting transmission. The infectiousness of asymptomatic individuals infected with *Leishmania*, markers for progression to VL disease, the role of domestic animals in transmission, and the potential of PKDL as a reservoir for infection need to be better understood [92, 125–127]. The implications of HIV coinfection with VL for treatment failure and relapse, transmission dynamics, and development of parasite resistance to drugs need to be studied further, and strategies need to be developed and tested as appropriate [42]. The development of innovative approaches to impair infection through early case detection and treatment, particularly in remote or previously nonendemic areas, as well as vector surveillance systems, new methods to measure transmission, mathematical transmission modelling to measure progress post elimination, xenodiagnostic studies to measure reservoir potential, new noninvasive antigen-based diagnostic tools [41], better treatment of PKDL, and surveillance for drug resistance are some of the urgent research priorities for the immediate future [122, 128]. DWL as an option for vector control needs to be further explored, as well as other alternatives that can be applied by communities themselves, such as insecticidal paint or LLIN combined with other affordable “do-it-yourself” measures with appropriate support. Further research is needed on insecticide resistance monitoring, sandfly breeding and feeding habits, and the impact of IRS on transmission of VL between the host and vector [129, 130]. Continuing investment in translational research from the bench to the bedside to public health is imperative to block transmission and prevent a resurgence of VL in the future.

**Key learning points**

- TDR engaged with national policy makers, scientists, and clinicians in the development and validation of strategies for elimination of VL in the Indian subcontinent.
- Linkage with the national programmes from the conception stage to identify and prioritize research needs facilitated the uptake of evidence-based interventions into national programme and policy.
- TDR’s stewardship role in supporting intervention research, technical advice, training, and policy contributed to the elimination of VL in the Indian subcontinent.

**Top five papers**

1. Cunningham J, Hasker E, Das P, El Safi S, Goto H, Mondal D, et al. A global comparative evaluation of commercial immunochromatographic rapid diagnostic tests for visceral leishmaniasis. Clinical Infect Dis: an official publication of the Infectious Diseases Society of America. 2012;55(10):1312–1319.
2. Bhattacharya SK, Sinha PK, Sundar S, Thakur CP, Jha TK, Pandey K, et al. Phase 4 trial of miltefosine for the treatment of Indian visceral leishmaniasis. J Infect Dis. 2007;196(4):591–598.
3. Mondal D, Alvar J, Hasnain MG, Hossain MS, Ghosh D, Huda MM, et al. Efficacy and safety of single-dose liposomal amphotericin B for visceral leishmaniasis in a rural public hospital in Bangladesh: a feasibility study. Lancet Glob Health. 2014;2(1):e51-e57.
4. Singh SP, Hirve S, Huda MM, Banjara MR, Kumar N, Mondal D, et al. Options for active case detection of visceral leishmaniasis in endemic districts of India, Nepal and Bangladesh, comparing yield, feasibility and costs. PLoS Negl Trop Dis. 2011;5(2):e960.
5. Joshi AB, Das ML, Akhter S, Chowdhury R, Mondal D, Kumar V, et al. Chemical and environmental vector control as a contribution to the elimination of visceral leishmaniasis on the Indian subcontinent: cluster randomized controlled trials in Bangladesh, India and Nepal. BMC Med. 2009;7:54.

## Supporting information

S1 Appendix WHO TDR–funded and/or WHO TDR–supported drug development research towards elimination of visceral leishmaniasis in the Indian subcontinent. Abbreviations: TDR, Special Programme for Research and Training in Tropical Diseases; WHO, World Health Organization.
(DOCX)
