# Supplementary material for: Towards elimination of visceral leishmaniasis in the Indian subcontinent—Translating research to practice to public health
Source: PLoS Negl Trop Dis. 2017 Oct 12;11(10):e0005889. doi: 10.1371/journal.pntd.0005889 (PMC5638223; doi:10.1371/journal.pntd.0005889)
Supplement: S1 Appendix — Abbreviations: TDR, Special Programme for Research and Training in Tropical Diseases; WHO, World Health Organization. (DOCX) [file pntd.0005889.s001.docx]

**Appendix**: WHO TDR funded / supported drug development research towards elimination of visceral leishmaniasis in the Indian sub-continent

| **Author**  **Year**  **Country**  **Reference** | **Year / extent of WHO**  **engagement** | **Study design**  **Subjects**  **Drug**  **Sample size** | **Results** | **Conclusion** |
| --- | --- | --- | --- | --- |
| ***Miltefosine*** | | | | |
| Jha  1999  India  [12] | Funding  Authorship | Phase II dosage trial (Miltefosine – Asta Medica) in 120 adults with VL  Arm 1: 50mg/d x 6wk  Arm 2: 50mg/d x 1wk, 100mg/d x 3wk  Arm 3: 100mg/d x 4wk  Arm 4: 100mg/d x 1wk, 150mg/d x 3wk | Initial parasite cure: 100%;  Clinical cure at 6mo:  Arm 1: 93%  Arm 2: 93%  Arm 3: 97%  Arm 4: 97%  Overall: 95% (95%CI: 89-98%) | Miltefosine effective treatment option |
| Sundar  2002  India  [19] | 1999-2000  Funding | Phase III comparative trial Miltefosine (Zentaris), Amph B (Fungizone) in 398 adults with VL  Miltefosine: 100mg/d x 28d – 299 pts  Amph B: 1mg/alt d x 15 – 99 pts | Initial parasite cure:  Miltefosine – 98%  Amph B – 99%  Clinical cure at 6mo:  Miltefosine – 94%  Amph B – 97% | Oral Miltefosine as effective as intravenous amphotericin B |
| Sundar  2003  India  [20] | 1999-2000  Funding | Phase I/II dosage trial (Miltefosine – Zentaris) in 39 children with VL  Arm 1: 1.5mg/kg/d x 28d – 21 pts  Arm 2: 2.5mg/kg/d x 28d – 18 pts | Initial parasite cure:  Arm 1 – 100%  Arm 2 – 100%  Clinical cure (ITT) at 6mo:  Arm 1 – 90%  Arm 2 – 83% | Oral Miltefosine is safe and effective in children with VL |
| Bhattacharya  2004  India  [21] | 2001-02  Funding  Authorship | Single arm phase I/II trial (Miltefosine – Zentaris)  in 80 children with VL  2.5mg/kg/d x 28d – 80 pts | Initial parasite cure – 99%  Final clinical cure – 94% | Oral Miltefosine is safe and effective in children with VL |
| Bhattacharya  2007  India  [24] | Funding  Monitoring  Drug supply | Single arm phase IV trial (Miltefosine – Zentaris)  in 704 adult and 428 children with VL  50/100mg (adults), 2.5mg/kg x 28d | Initial parasite cure (ITT) – 93.2%  Final clinical cure (ITT) – 82% | Miltefosine is effective for VL treatment in an outpatient setting |
| ***Liposomal amphotericin*** | | | | |
| Thakur  1996  [13]  Berman  1998  India  [14] | Funding  Authorship | Phase II dosage trial (L-AmB – NeXstar)  in 30 adults with VL  Arm 1: 2mg/kg x d1-d6, d10 (total 14mg/kg)  Arm 2: 2mg/kg x d1-d4, d10 (total 10mg/kg)  Arm 3: 2mg/kg x d1, d5, d10 (total 6mg/kg) | Final clinical cure (12mo): 100%  One patient in 2^nd^ arm died of unrelated cause | Liposomal amphotericin B highly efficacious for VL treatment, well tolerated |
| Sundar  2004  India  [22] | Funding  2001 | Phase III comparative trial (Fungizone – Sarabhai, L-AmB – Gilead, Abelcet – Liposome Co.) in 153 adults and children with VL  Arm 1: Fungizone 1mg/kg eod x 15  Arm 2: L-AmB 2mg/kg x 5d  Arm 3: Abelcet 2mg/kg x 5d | Final clinical cure (6mo):  Fungizone – 96%  L-AmB – 96%  Abelcet – 92%  Liposomal amphotericin better tolerated | Liposomal formulations of amphotericin preferred as better tolerated, shorter therapy and less hospitalization costs |
| Bern  2006  [23] | Funding  Authorship | WHO expert consultation to develop guidelines for liposomal amphotericin B treatment of VL for national VLEP | High efficacy, low toxicity in immunocompetent patients;  Access poor due to high cost; | 1^st^ line of treatment for VL in areas where of drug resistance and VL co-infection with HIV;  Need for preferential pricing for national VLEP |
| Mondal  2014  Bangladesh  [25] | 2012  Funding  Authorship | Single arm phase III trial (L-AmB – Gilead) in 300 VL adults and children  L-AmB 10mg/kg x d1 | Initial clinical cure (1mo): 87%  Final clinical cure (6mo): 97%  Well tolerated | Single dose L-AmB treatment for VL feasible at primary health centre level |
| Maintz  2014  Bangladesh  [29] | 2012  Funding  Authorship | Feasibility, acceptability study to use single dose L-AmB (Gilead)  FGD with VL patients, KII with program managers, health staff | Strong political commitment;  High level of satisfaction among VL patients, health staff; Less income loss, better experience, perceived benefits with single dose L-AmB than with Miltefosine; | Single dose L-AmB preferred option as 1^st^ line treatment for VL in Bangladesh;  Health infrastructure for drug storage, ancillary supplies for drug administration needs to be strengthened |
| ***Paramomycin*** | | | | |
| Jha  1998  India  [15] | 1996  Funding  Authorship | Phase II dosage trial (PM, SSG) in 120 adults and children with VL in 4 study arms  PM 12, 16, 20mg/kg x 21d  SSG 20mg/kg x 28d | Final clinical cure (6mo):  PM 12mg/kg: 77%  PM 16mg/kg: 93%  PM 20mg/kg: 97%  SSG 20mg/kg: 63% | PM 16, 20mg/kg x 21 d effective 1^st^ line for VL treatment in areas of resistance to antimonial |
| Thakur  2000  India  [17] | 1996  Funding  Authorship | Phase II dosage trial (PM, SSG) in 120 adults and children with VL in 4 study arms  PM 12, 16, 20mg/kg x 21d  SSG 20mg/kg x 28d | Final clinical cure (6mo):  PM 12mg/kg: 90%  PM 16mg/kg: 88.9%  PM 20mg/kg: 86.2%  SSG 20mg/kg: 69% | PM to replace SSG as 1^st^ line for VL treatment in areas of antimonial resistance |
| ***Combination therapy*** | | | | |
| Thakur  1995  India  [16] | Authorship | Phase II dosage trial (PM, SSG) in 96 adults and children with VL each in 3 successive studies with 3 arms each  Study 1:PM 12mg/kg + SSG (20, 10, 5mg/kg) x 21d  Study 2:PM 6mg/kg + SSG (20, 10, 5mg/kg) x 21d  Study 3 (discontinued):PM 3mg/kg + SSG (20, 10, 5mg/kg) x 21d | Initial parasite cure:  Study 1: PM 12mg/kg  + SSG 20mg/kg – 88%  + SSG 10mg/kg – 71%  + SSG 5mg/kg – 72%  Study 2: PM 6mg/kg  + SSG 20mg/kg – 69%  + SSG 10mg/kg – 50%  + SSG 5mg/kg – 46% | Combination of PM (12mg/kg) and SSG (20mg/kg) x 21d more effective and safe than SSG (20mg/kg) x 40d for VL treatment in areas of antimonial resistance |
| Thakur  2000  India  [18] | 1996  Funding  Authorship | Phase II dosage trial (PM, SSG) in 150 adults and children with VL in 3 study arms  Arm 1:PM 12mg/kg + SSG 20mg/kg x 21d  Arm 2:PM 18mg/kg + SSG 20mg/kg x 21d  Arm 3: SSG 20mg/kg x 28d | Final clinical cure:  Arm 1 – 92.3%  Arm 2 –93.8%  Arm 3 – 53.13% | Combination of PM and SSG for 21d is safe, effective low-cost alternative to SSG alone for 28d |
| Sundar  2008  India  [26] | 2006-07  Authorship | Phase II dosage trial (L-AmB – Gilead, Miltefosine – Zentaris) in 226 adults with VL in 5 study arms  L-AmB (5mg/kg x d1) alone (arm 1), or combined with Miltefosine x 10d (arm 2) or 14d (arm 3); or L-AmB (3.75mg/kg x d1) with Miltefosine x 14d (arm 4) or 7d (arm 5) | Final clinical cure (9mo):  L-Amb (5mg/kg) - 91%  L-Amb (5mg/kg + miltefosine x 10dy) - 98%  L-Amb (5mg/kg + miltefosine x 14dy) - 96%  L-Amb (3.75mg/kg + miltefosine x 14dy) - 96%  L-Amb (3.75mg/kg + miltefosine x 7dy) - 98% | Single dose L-AmB at reduced dosage combined with miltefosine for shorter duration is effective for VL treatment |
| Sundar  2011  India  [27] | 2008-09  Authorship | Phase II short duration trial (Amph B – Piramal, L-AmB – Gilead, Miltefosine – Paladin, PM – Gland) in 634 adults and children with VL in 4 study arms  Amph B (1mg/kg x 15eod) alone (arm 1), or L-AmB (5mg/kg x d1) combined with Miltefosine (arm 2) or PM (11mg/kg) (arm 3); or Miltefosine combined with PM (11mg/kg) (arm 4) | Final clinical cure (6mo):  Arm 1 : AmphB x 15 eod - 93%  Arm 2: L-Amb + Miltefosine x 7d - 97.5%  Arm 3: L-Amb + PM x 10d - 97.5%  Arm 4: Miltefosine + PM - 98.7% | All combinations more effective than monotherapy with AmphB |
| Sundar  2011  India  [28] | 2007-09  Funding  Authorship | Single arm phase III trial (L-AmB – Gilead, Miltefosine – Paladin) in 135 adults and children with VL  L-AmB (5mg/kg x d1) + Miltefosine x 14d | Final clinical cure (6mo):  ITT: 91.9% | Single dose L-AmB combined with Miltefosine for 14d is well tolerated, effective treatment for VL |
| ***Reviews*** | | | | |
| Olliaro  2005  India  [30] | Authorship | Review of treatment options for VL at a time of increasing drug resistance to SSG;  53 studies (SSG-13, Miltefosine-7, AmphB-18, L-AmB-6, L-AmphB-8, pentamidine-7, PM-5) | Pentamidine – toxic, declining efficacy;  AmphB – effective, long duration, toxic;  L-AmB – effective, safe, expensive;  Miltefosine – effective, potential for drug resistance, side-effects;  PM – effective, safe, not yet registered | Need for supervised deployment and use of limited armamentarium of drugs;  R&D for combination therapy to reduce cost, shorten duration, delay resistance;  Need for active pharmacovigilance |
| Alvar  2006  [31]  Croft  2011  [32] | Authorship | Review / commentary on drug treatment options for VL | Opportunities: Single dose L-Amb and combination therapy;  Challenges: Treatment options for PKDL and VL co-infection with HIV | Newer drugs better but not ideal;  R&D must continue for safe, cost-effective treatment options;  Need treatment strategies to delay or prevent drug resistance |
| Olliaro  2010  ISC  [33] | Authorship | Review of efficacy of combination therapy for VL | High efficacy (>95%) with:  L-AmB + Miltefosine or PM x 7d, or  Miltefosine + PM x 10d | No combinations therapy trials in Bangladesh, Nepal  Currently no options for fixed dose combination treatment for VL |
| Van Griensven  2010  ISC  [34] | Authorship | Review of combination therapy options for VL | Combination therapy is more cost-effective – reduces treatment duration (8-10d), hospitalization burden, indirect costs, better compliance | Research needed to determine if combination therapy delays development of drug resistance |
| Kshirsagar  2011  ISC  [35] | Funding  Authorship | Review of pharmacovigilance for Miltefosine for VL treatment | National pharmacovigilance program exists in India, Nepal but not in endemic regions,  does not exist in Bangladesh | Need to set up monitoring of cohort events, prescription practices, drug supply and quality, pregnancy register |
| Matlashewski  2011  ISC  [36] | Authorship | Commentary on VL treatment options for national VLEP | Attack phase – Single dose liposomal amphotericin B at primary health centres;  Maintenance phase – combination therapy with Miltefosine and PM to ensure long term availability and protect against drug resistance | |

Note: L-AmB = Liposomal Ambisome ®, ISC = Indian sub-continent, AmphB = Amphotericin B, PM = Paramomycin, SSG = Sodium stibogluconate
